# 

**Published:** 1900-05-26

**Authors:** 


					/
THE HOSPITAL. "The standard of highest purity."?Lancet. May 26th, lf)00.
CADBURY'S If CADBURY'S
r.nnnA \1\. il'ii - cnr.nA
COGOA ^ <M^> COCOA
ABSOLUTELY POU, NO DRUGS OR ALKALIES USED.
No. 713) Vol. XXVIII. PSSSU3 Saturday,
May 26, 1900.
[S^scriptionTetms.J pRICE TWOPENCE.

				

